# Case Report: Ankle fracture caused by anterior-posterior horizontal force: a report of two cases

**DOI:** 10.3389/fsurg.2026.1962292

**Published:** 2026-10-16

**Authors:** Bang Dou, Jiaxun Zhao, Wenqian Ma, Junshuai Lu, Jiong Mei

**Affiliations:** 1 Department of Orthopedics, Songjiang Hospital Affiliated to Shanghai Jiao Tong University School of Medicine, Shanghai, China; 2 School of Mechanical and Automotive Engineering, Shanghai University of Engineering Science, Shanghai, China; 3 Department of Orthopedic Surgery, Shanghai Jiao Tong University Affiliated Sixth People’s Hospital, Shanghai, China

**Keywords:** ankle fracture, extension, flexion, horizontal violence, osteofascial compartment syndrome

## Abstract

**Background:**

Ankle fractures caused by violence in the anterior-posterior horizontal direction are very rare in clinical practice. Few literatures report on the characteristics and mechanisms of this type of fracture, which should be studied to guide clinical treatment.

**Case presentation:**

We presented two cases of ankle fractures caused by horizontal violence in the anterior-posterior direction, and these fractures presented a distinctive morphological pattern. After injury, the affected limbs showed significant swelling, and case 2 had developed osteofascial compartment syndrome, and immediate reduction and incision decompression had been performed. After the soft tissue swelling subsided, an open reduction and internal fixation surgery was performed. We fixed the tibial fractures first and then the fibular fractures, and fixed tibial fractures in anterior and medial biplane, which achieved good clinical efficacy.

**Conclusion:**

Ankle joint fracture caused by horizontal force in the anterior-posterior direction may be a flexion/extension injury, which is rare and unstable and needs further research on the mechanism of injury. This type of ankle fracture may be accompanied by osteofascial compartment syndrome, which needs careful examination and timely treatment.

## Introduction

Ankle fractures are one of the most common musculoskeletal injuries, with an incidence rate of 168.7 per 100,000 individuals (1), accounting for approximately 9% of all fractures (2). As we know, fracture classification serves as a crucial tool in clinical practice and research, and a good classification system should provide guidance for surgeons in terms of treatment and prognosis (3). There are many classifications of ankle fractures. Ashhurst and Bromer were the first to propose an etiological classification, which divided ankle fractures into four subtypes based on the nature of external forces: adduction, abduction, external rotation, and vertical compression (4). In 1950, Lauge-Hansen proposed the Lauge-Hansen classification (5) based on the mechanism of injury, which divided ankle fractures into four subtypes: supination/adduction, supination/external rotation, pronation/abduction, and pronation/external rotation. Over 90%–95% of x-ray films could be classified according to this classification, but there are still 8.8%–12.0% of cases that are not applicable (3, 6). Due to the complexity of the Lauge-Hansen classification, the purely descriptive Danis-Weber classification was proposed. In recent years, some new classifications based on ankle joint stability have been proposed too (7, 8).

This article reported two cases of ankle fractures caused by horizontal violence in the anterior-posterior direction, and these fractures presented a distinctive morphological pattern. Neither the Ashhurst and Bromer etiological classification nor the Lauge-Hansen classification was applicable for these fractures. Although these fractures could be categorized as Danis-Weber type C or AO/OTA 43-A, these two classifications were too general and cannot reflect the characteristics of this type of fracture well.

## Case presentation

### Case 1

A 55-year-old woman was hit head-on by a car and suffered high-energy damage to her left ankle. This was the first occurrence in the patient's clinical history. Physical examination showed that the patient's ankle joint was dorsiflexed at the distal end and mildly everted. Although the patient's left ankle had been swollen significantly, the left dorsalis pedis artery was still pulsating well. CT scan of the ankle joint showed a fibular fracture accompanied by a transverse fracture of the distal end of the tibia. The fibula presented as a comminuted oblique fracture about 1 cm above the tibiofibular syndesmosis, with tibial fracture ends angled backwards. Both ends of the tibia fracture were intact, and the posterior fracture line was very close to the articular surface (Figures 1A–D).

**Figure 1:**
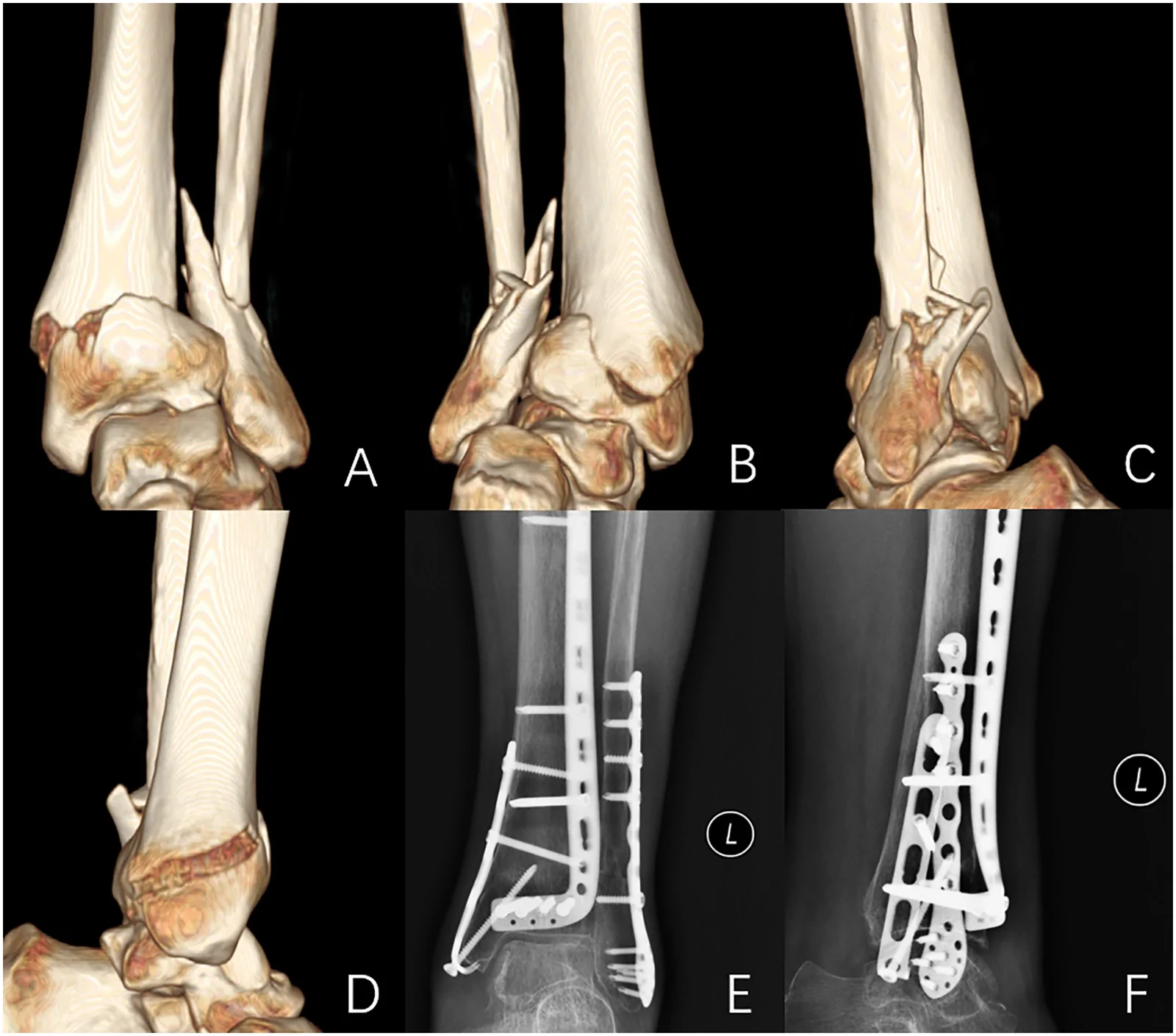
**(A–D)** The patient in case 1 was a 55-year-old female. CT three-dimensional reconstruction showed a Danis Weber type C fibular fracture, with a comminuted left fibular fracture and a left tibial fracture in two parts. The posterior tibial fracture line was close to the articular surface, while the anterior tibial fracture line was approximately horizontal, with dorsiflexion and angulation of the ankle joint. **(E,F)** Postoperative x-rays showed anatomical reduction of the fracture.

Emergency closed reduction and temporary external fixation with braces were performed. On the 8th day after fracture, soft tissue swelling subsided, and open reduction and internal fixation was performed. During the surgery, the patient was placed in a supine position, and an anterior-medial approach combined with a lateral approach to the ankle joint was adopted. We reduced and fixed the tibial fracture first, using an anterior lateral L-shaped steel plate and an inner thin steel plate, then reduced and fixed the fibula. The “HOOK” test had been performed during the operation and showed a good stability of the distal tibiofibular syndesmotic ligament.

On the second day post-surgery, patients engaged in isometric muscle contraction exercises, along with mobilization of their toes and knee joints. By the third day post-surgery, dorsiflexion exercises for the ankle joint were commenced. Postoperative x-ray examination showed the fracture was reduced excellently according to the Burwell-Charnley radiographic criteria (Figures 1E,F). The patient was discharged one week after surgery, and during a follow-up examination one month after surgery, the ankle joint mobility was good, with no infection. Four months after surgery, the fracture healed. One year after surgery, functional outcomes were evaluated using the American Orthopaedic Foot and Ankle Society (AOFAS) Ankle-Hindfoot Scale, which showed the AOFAS score was 95. And there was no traumatic ankle arthritis.

### Case 2

A 22-year-old male was rear ended by a car while riding an electric scooter 2 h before admission, and his right ankle joint was injured. He was sent to our emergency orthopedic department. Physical examination showed that the patient had distal plantarflexion and mild eversion of the ankle joint. There was a 3 cm long wound on the inner side of the ankle joint. The patient's right dorsal artery couldn't be reached, the right calf and right foot were significantly swollen, and the skin temperature had decreased. The patient complained of numbness in the right foot.

CT scan of the ankle joint showed a Danis Weber C-type ankle fracture. The fibula was fractured in a long oblique shape about 2 cm above the tibiofibular joint, and the tibia fractured into two parts. The anterior fracture line was very close to the joint surface, and the tibia and fibula fractures were angled forward (Figures 2A–C). There were no fractures at the proximal tibia and proximal fibula (Figure 2D).

**Figure 2:**
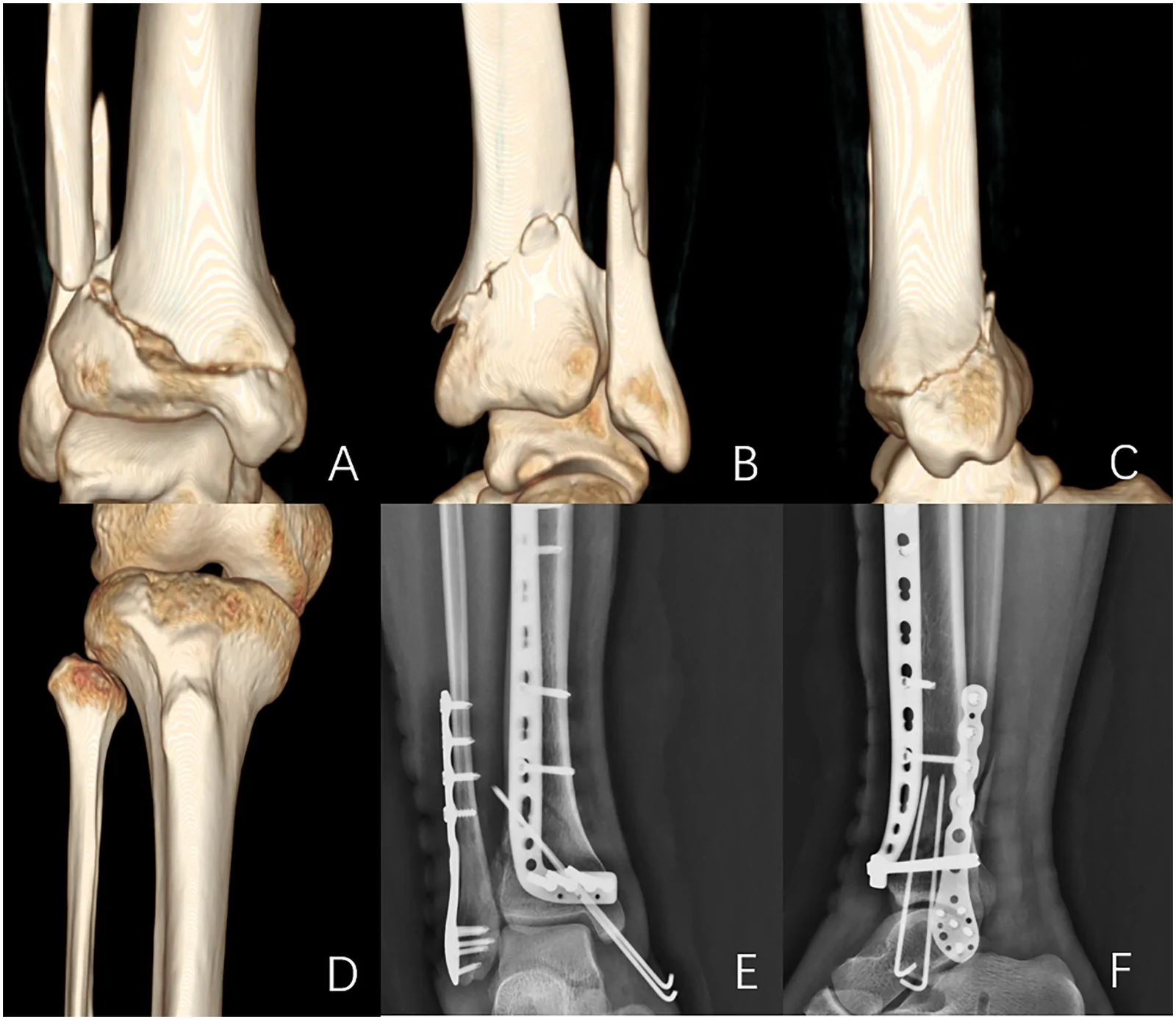
**(A–C)** the patient in case 2 was a 22-year-old male. CT three-dimensional reconstruction revealed a Danis Weber type C fibular fracture, with the right tibia fractured into two parts. The anterior tibial fracture line was approximately transverse, close to the ankle joint surface, with the ankle joint in plantarflexion and eversion. **(D)**There were no fractures at the proximal end of the fibula. **(E,F)** Postoperative x-rays showed anatomical reduction of the fracture.

We diagnosed the patient with osteofascial compartment syndrome and performed emergency debridement, incision and decompression, closed reduction and external fixation. All four leg compartments of anterior, lateral, superficial posterior and deep posterior were decompressed during emergency fasciotomy. After sufficient decompression, the pulse of the dorsalis pedis artery was still not palpable. Emergency arterial color Doppler ultrasound examination was performed, and although the right dorsalis pedis artery was partially displayed, the blood flow in the displayed segment was unobstructed. We monitored the pulse of the patient's dorsalis pedis artery closely, and the dorsalis pedis artery pulsated very strongly the next day.

The fasciotomy wounds were covered with Vaseline, and antibiotics were administered to prevent infection after the surgery. Thirteen days later, the soft tissue swelling subsided and an open reduction and internal fixation surgery was performed. Similarly, the ankle joint anterior midline approach and lateral approach were adopted. During the operation, soft tissue entrapment was observed on the lateral of the tibial fracture, which had been relieved. We reduced and fixed the tibial fracture first, using an anterior lateral L-shaped steel plate and two 2.0 mm diameter Kirschner wires, then reduced and fixed the fibula. And a good stability of the distal tibiofibular syndesmotic ligament had been confirmed by the “HOOK” test too.

Postoperative x-ray examination showed anatomical reduction according to the Burwell-Charnley radiographic criteria (Figures 2E,F). The ankle joint mobility was good one month after surgery, and the fracture healed three months after surgery. The AOFAS score was 99 at the last follow-up, and there was no traumatic ankle arthritis.

## Discussion

Currently, the commonly used classifications for ankle fractures include Lauge-Hansen classification, Ashhurst classification, Danis Weber classification, and AO/OTA classification, as well as some special types of fractures, such as Logsplitter fracture (9–11), Bosworth fracture (12, 13), Maisonneuve Fracture (14), and so on.

Although these two cases could be classified as Danis-Weber type C or AO/OTA 43-A, these two classifications were proposed according to the fracture location, which were purely descriptive classification, and too general, and couldn’t reflect the characteristics of these two fractures well.

These two cases reported in this article were a special type resulting from anterior-posterior violence. They were so special that the tibia just fractured in two parts, and the tibial fracture line was almost transverse and located at the extreme distal end of the tibia, which were different from other extra-articular distal tibia fractures in which the fracture lines of the tibia were generally oblique and had a certain distance from the distal articular surface of the tibia (15, 16), and the distal tibial articular surface remained intact, which was different from intra-articular fracture, such as Pilon fracture caused by vertical violence (17).

Based on the direction of angular deformation at the fracture site, it could be divided into two opposite types: anterior angulation and posterior angulation, which suggested that the mechanisms might be completely opposite.

### Possible mechanism of injury

#### Case 1

We utilized CT data of the fracture for 3D reconstruction and virtual reduction and then analyzed the fracture mechanism through manual schematic kinematic illustrations derived from CT models (Figure 3). Thus, we speculated that the patient with this type of fracture may bend the upper body forward during a direct impact from the rear to the upper body or a forward inertia effect during deceleration, while the ankle remained relatively stationary, resulting in an anterior flexion injury to the ankle, which liked the Chance fracture of the spine (18, 19). We speculated that the mechanism of injury of this case may be an anterior flexion injury.

**Figure 3:**
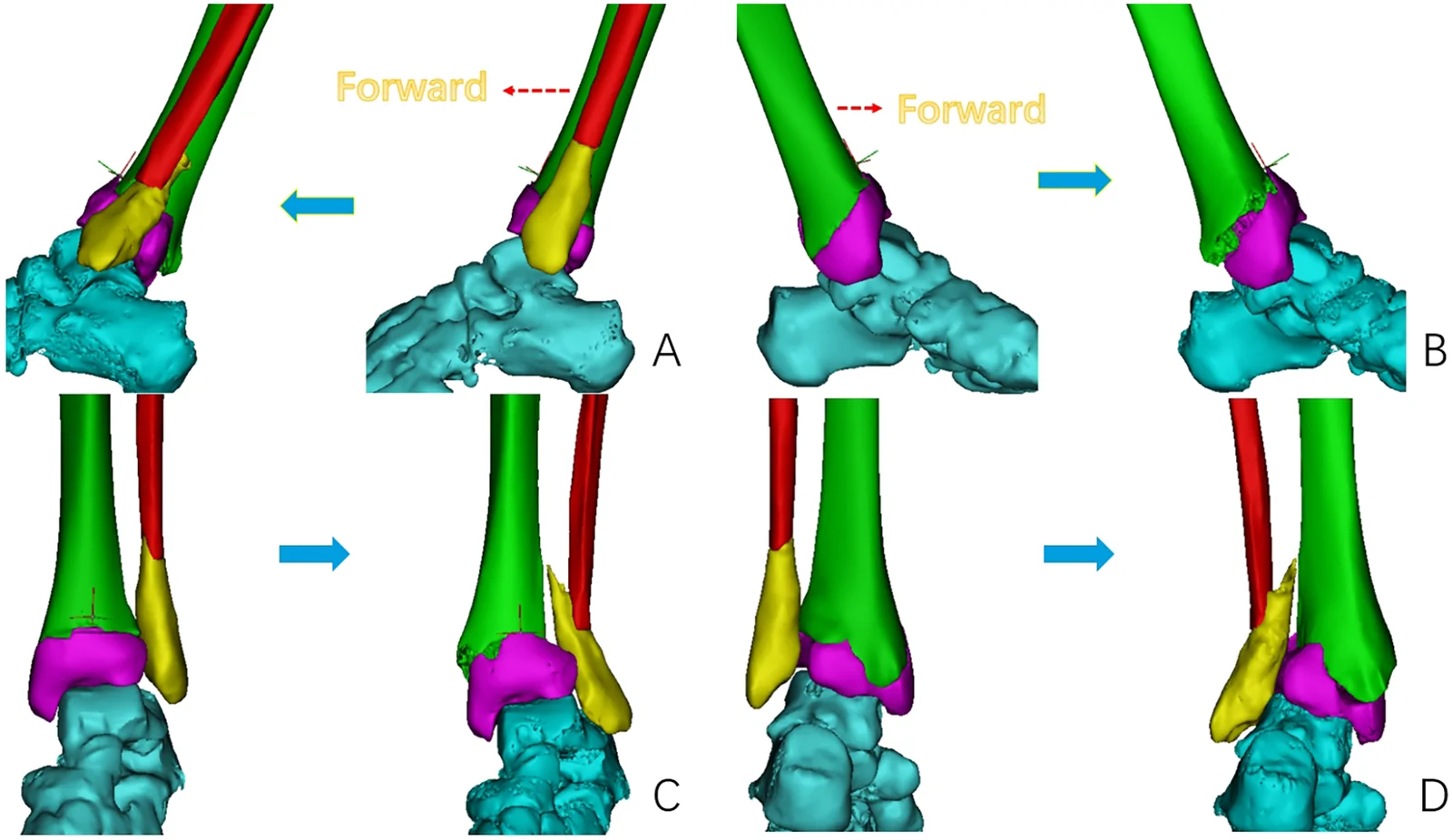
**(A–D)** Simulate the process of ankle fracture in case 1 with CT three-dimensional images in different observation views.

The patient of case 1 recalled that she was hit head-on by a car while driving an electric scooter, her foot and ankle were stuck, and the upper part of the body fell forward. We speculated that after the collision, the electric scooter decelerated forward, the foot and ankle were stuck and decelerated too, then the upper part of the patient's body fell forward due to inertia, which was consistent with our analysis of the mechanism of this injury.

#### Case 2

Similarly, we utilized CT data of the fracture for 3D reconstruction and virtual reduction and then analyzed the fracture mechanism through manual schematic kinematic illustrations derived from CT models (Figure 4). Thus, we speculated that the patient with this type suffered an anterior-posterior violence to the upper body or a backward inertia effect during acceleration, then the upper part of the body fell backward while the ankle remained relatively stationary, resulting in a posterior extension injury of the ankle, just like a reverse Chance fracture. We speculated that the mechanism of injury of this case may be a posterior extension injury.

**Figure 4:**
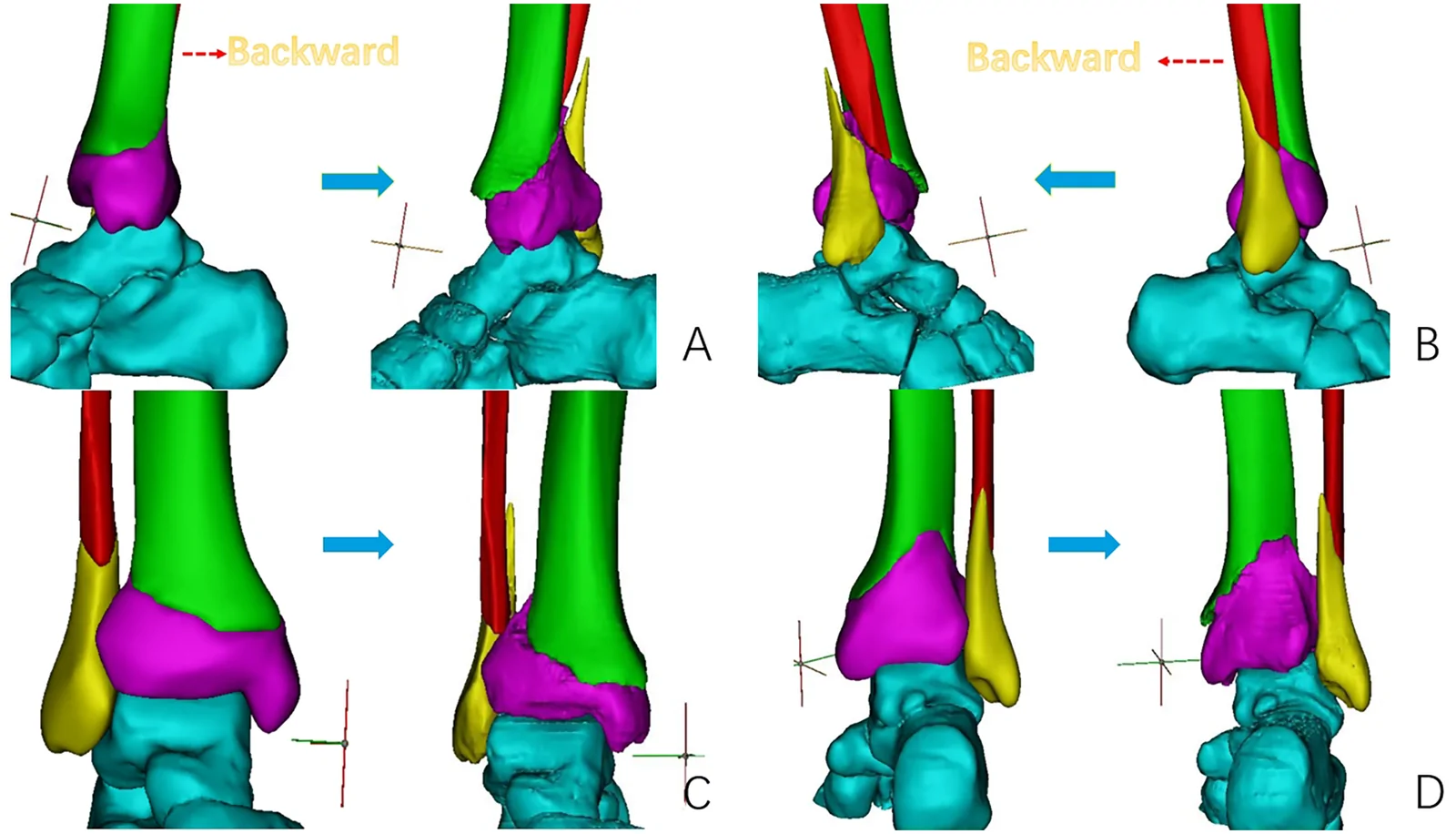
**(A–D)** Simulate the process of ankle fracture in case 2 with CT three-dimensional images in different observation views.

The patient of case 2 recalled that he was rear ended by a car while driving an electric scooter and the upper part of the body fell backward. We speculated that after the collision, the electric scooter accelerated forward, while the upper part of the patient's body fell backward due to inertia, which was consistent with our analysis of the mechanism of this injury.

### Characteristics of fractures

In fractures caused by horizontal force in the anterior-posterior direction, the fibular fracture was located above the distal tibiofibular syndesmosis. And the tibia fractured into two parts, with the fracture line on the tension side being approximately horizontal and being closer to the ankle joint surface. It resulted in the posterior malleolar fracture part being thinner than the anterior malleolar fracture part in anterior flexion type ankle fracture and the anterior malleolar fracture part being thinner than the posterior malleolar fracture part in posterior extension type ankle fractures.

Due to these ankle fractures caused by violence in the anterior-posterior horizontal direction without rotation, preoperative CT scans showed good curvature and spacing of the inferior tibiofibular joint, and intraoperative “HOOK” testing showed the syndesmosis was tight, all of these meant the ligament of the inferior tibiofibular joint might be intact in this type of fracture.

The patient of case 2 had developed an osteofascial compartment syndrome, with no fracture at the high fibular position. Unfortunately, vascular ultrasound or angiography was not performed to mention compartment pressure and angiographic evidence as the condition was quite urgent at the time and required an emergency incision and decompression. We had to analyze it from the perspective of imaging that there might be two causes for this. First, during the valgus angular displacement of the patient, the proximal tibial fracture fragment shifted medially, while the proximal fibular fracture fragment was blocked by the distal tibialfracture fragment (Figures 5A–C). It resulted in a tear of the tibiofibular interosseous membrane, significant separation atthe proximal ends of the tibial and fibular fractures, and massive hemorrhage within the compartments. Second, due to the anterior angulation of the fracture ends, the anterior tibial artery was subjected to traction andcompression (Figure 5D), which might be another important reason for the occurrence of osteofascial compartment syndrome.

**Figure 5:**
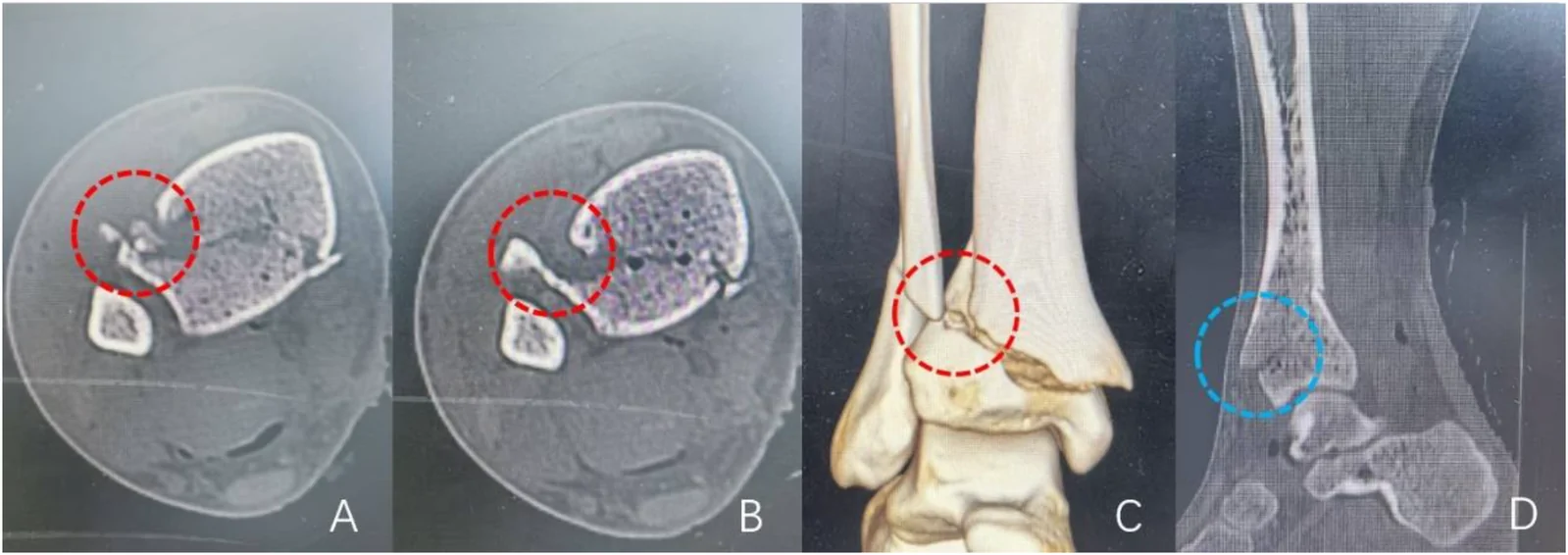
**(A–C)** the red circles indicated that the proximal fibular fracture was obstructed by the distal tibial fracture end, resulting in significant separation at the proximal end of the tibiofibular fracture. **(D)** The blue circle showed that the ankle joint fractured end was angulated forward, and the anterior tibial artery might be compressed.

### Treatment

Since the tibial fracture line was too close to the ankle joint and the distal fracture fragment of tibia was too thin, biplane fixation was required for tibia treatment.

In Case 1, we used an anterolateral plate and a medial plate rather than a posterior anti-glide plate because the posterior fracture line was too close to the distal end of the tibia, the distal screws of the posterior plate cannot fix the distal fracture effectively, and if distal screws are used, they may even be inserted into the ankle joint.

In Case 2, Since the anterior tibial fracture line was too close to the distal end, the connection between the medial malleolus and the anterior body of the distal fracture fragment was little, the distal screw of the anterolateral plate cannot fix the distal fracture effectively. To enhance the stability of the distal tibial fracture and prevent medial malleolus fractures during early functional exercises, we inserted two Kirschner wires into the contralateral cortex percutaneously and removed the two Kirschner wires 3 months after surgery.

It should be emphasized that in the case of posterior extension type of ankle fracture, due to the angle towards the front after fracture and the soft tissue in the front was very thin, the anterior tibial artery was prone to compression. Therefore, timely reduction was needed. If compartment syndrome occurs, immediate reduction and incision decompression should be performed.

## Conclusions

Ankle joint fracture caused by horizontal force in the anterior-posterior direction maybe a rare and flexion/extension injury, which is unstable and requires biplane fixation for tibia. This type of ankle fracture may be accompanied by osteofascial compartment syndrome, careful examination and timely treatment are strongly recommended. Further research such as finite element analysis (FEA) and biomechanical loading test of cadaver specimens and more cases are needed for the mechanism of injury.

## Data Availability

The original contributions presented in the study are included in the article/Supplementary Material, further inquiries can be directed to the corresponding author.
